# From bubbling to boiling over: a meta-ethnography of the process towards and during crisis from the perspectives of persons living with dementia, informal carers and healthcare professionals

**DOI:** 10.1093/ageing/afaf383

**Published:** 2026-01-24

**Authors:** Stella Thissen, Eefje Sizoo, Martin Smalbrugge, Debby L Gerritsen, Marieke Perry

**Affiliations:** University Knowledge Network for Older Adult Care Nijmegen (UKON), Department of Primary and Community Care, Radboud University Medical Center, Nijmegen, GE, Netherlands; Radboudumc Alzheimer Center, Radboudumc, Nijmegen, GE, Netherlands; Ageing and Later Life, Amsterdam Public Health Research Institute, Amsterdam, North Holland, Netherlands; Ageing and Later Life, Amsterdam Public Health Research Institute, Amsterdam, North Holland, Netherlands; Department of Medicine for Older People, Amsterdam UMC Location VUmc, Amsterdam, Noord-Holland, Netherlands; University Knowledge Network for Older Adult Care Nijmegen (UKON), Department of Primary and Community Care, Radboud University Medical Center, Nijmegen, GE, Netherlands; Radboudumc Alzheimer Center, Radboudumc, Nijmegen, GE, Netherlands; University Knowledge Network for Older Adult Care Nijmegen (UKON), Department of Primary and Community Care, Radboud University Medical Center, Nijmegen, GE, Netherlands; Radboudumc Alzheimer Center, Radboudumc, Nijmegen, GE, Netherlands

**Keywords:** dementia, crisis, emergency, healthcare professionals, informal caregivers, qualitative research, older people

## Abstract

**Background:**

Crisis admissions in dementia care are increasing, often leading to negative outcomes for people with dementia, their informal caregivers and healthcare professionals. Crises arise from a complex interplay of health, behavioural, social and environmental factors.

**Objective:**

This systematic review of qualitative studies, using a meta-ethnographic approach, explores the process leading up to and unfolding during crises.

**Methods:**

Five databases were searched for studies published between January 2000 and September 2023. Study selection involved AI-assisted screening (ASReview), followed by manual review and quality appraisal using the Joanna Briggs Institute checklist. Data synthesis was guided by the Strauss and Corbin qualitative framework.

**Results:**

Nineteen studies, mainly reflecting the perspectives of informal caregivers and healthcare professionals, were included in the analysis. The core phenomenon identified is the mechanism in which professionals, persons with dementia and informal caregivers are constantly balancing between safety and autonomy, triggered by disruptions to a previously stable situation. Two contextual factors influence this process: a proactive, collaborative attitude among healthcare professionals, and a healthcare system that often acts as a push system, limiting flexibility and responsiveness.

**Conclusions:**

These findings highlight the need for collaborative care approaches to prevent or manage crises more effectively, offering valuable insights for practice and policy improvements.

## Key Points

- Professional and informal caregivers balance safety and autonomy during dementia crises.
- Proactive and collaborative professionals can help prevent crisis situations.
- System-level barriers hinder crisis prevention and response in dementia care.

## Introduction

Crisis situations in dementia care are increasingly common, resulting in negative health outcomes and stress for persons with dementia, their informal caregivers and healthcare professionals [1, 2]. This increase is partly attributed to population aging, with the global number of persons with dementia expected to rise from 57.4 million in 2019 to 152.8 million in 2050 [3]. In line with international trends of aging in place [4, 5], Dutch policy strongly encourages older people, including those with dementia, to live at home as long as possible [6]. While this aligns with preferences for home-based care, it also increases the risk of crises due to declining health, dementia symptoms, hazardous home environments and caregivers’ limited capacity to manage complex care needs [7]. A crisis is defined as a potentially dangerous situation requiring immediate intervention to prevent further deterioration [8]. Such events frequently result in emergency department (ED) visits and unplanned hospital or nursing home admissions [9, 10], which can cause functional decline, distress and increased risk of delirium in persons with dementia [11, 12]. Caregivers may also experience emotional stress, including feelings of loss [2, 13, 14]. Despite scarce evidence supporting that crisis intervention teams [15] and care coordination models [16, 17] may mitigate crises, two systematic reviews found insufficient evidence to support consistent reductions in hospital admissions [18, 19].

Given the anticipated increase in crisis situations, and the lack of effective interventions, emergent understanding of how crises develop and how to mitigate and manage crises is essential. Existing literature identifies contributing factors such as increased vulnerability, physical health issues, neuropsychiatric symptoms, caregiver burden and environmental hazards [9, 20]. These overlap with known risk factors for acute healthcare utilization, such as comorbidities, previous hospitalizations and behavioural disturbances [21–23]. However, crises often result from a complex interaction of health, behavioural, social and environmental factors [24]. Understanding this dynamic interplay is crucial for developing timely and targeted interventions.

Previous reviews by Vroomen et al. and Hopkinson et al. have studied the definition of crisis and the causes, presentation and management of crisis, respectively. The framework of Vroomen et al. highlighted the cyclical nature of the crisis process [8]. Hopkinson et al. presented findings in three phases: before, during and after crisis [24]. This relatively linear overview of crisis needs further exploration of the mechanisms in this process to provide actionable insights for informal caregivers and primary care professionals. Moreover, since the publication of Hopkinson et al., which covered literature up to 2019, interest in crisis reduction has grown and many potentially relevant studies have been published, which are likely to provide additional insights.

Building on the existing reviews, our systematic review therefore aims to comprehensively deepen the understanding of the lead-up and management of the crisis by uncovering the key elements in the process and explore mechanisms of their interrelations.

## Methods

### Study design

We performed a systematic review of qualitative studies taking a meta-ethnographic approach. Meta-ethnography is a method of synthesizing qualitative findings from multiple studies by interpreting and translating key concepts to generate new insights, in contrast to Hopkinson’s more exploratory mixed-methods approach. It enables the development of analytical insights rather than merely describing a phenomenon [25]. The PRISMA guidelines for systematic reviews were followed [26].

### Search strategy

The following databases were searched for relevant literature: Medline/PubMed, EMBASE, The Cochrane library, CINAHL and PsycINFO. The search was limited to articles between January 2000 and September 2023, because older literature may not reflect current care practices. Additionally, reference lists of eligible articles were screened for relevant articles. As the research question was exploratory, a sensitive search strategy was chosen based on search terms for ‘dementia’ and ‘crisis.’ Crisis was defined previously as ‘a process where a stressor causes an imbalance requiring an immediate decision to be made which leads to a desired outcome and therefore a resolution of the crisis. If the crisis is not resolved, the cycle continues’ [8]. We operationalized crisis based on terms describing acute actions, such as hospital admissions, best reflecting the immediate decision. This led to the following search terms: emergency, crisis, hospitalization, acute admission, nursing home admission, patient admission and emergency medical services. The exact search terms for every database can be found in Appendix 1. During the selection process, the scope narrowed to qualitative studies of this complex process. Table 1 shows the inclusion and exclusion criteria of the review.

**Table 1:** Eligibility criteria

Eligibility criteria	
Inclusion	Studies that include persons with a diagnosis of any type of dementia.
	Studies about the process of crisis situations or interventions aiming to reduce crises. This includes the lead-up to a crisis situation, the management of an occurring crisis situation and how a crisis situation is resolved.
	Studies about crisis situations at home.
	Studies including the perspectives of persons living with dementia, informal carers and healthcare professionals.
	Primary qualitative research.
	Published after 2000.
Exclusion	Studies about crisis situations that are not caused by dementia.
	Studies solely describing the management of a crisis in hospital.
	Quantitative research, case studies, conference abstracts and reviews.

### Study selection

Study records identified through the search strategy were imported to EndNote reference management software [27] and duplicates were removed. Titles and abstracts of the articles were screened in two phases. First, ASReview was employed to assess the relevance of articles. ASReview uses artificial intelligence to speed up the literature screening by training a model using the screening decisions of a human reviewer. This enabled us to efficiently screen the extensive volume of literature anticipated from our exploratory and sensitive search strategy [28]. The model presents the reviewer with references considered highly relevant, based on articles added by the reviewer as prior knowledge. Afterwards, it continuously reorders the articles based on the screening decisions of the reviewer. In this way, the reviewer can stop screening without having seen every article. We used the stopping criterion that 100 consecutive articles were marked as ‘irrelevant’ [29]. The review screening was conducted in duplicate, with one assessment completed by ST and the other by RB and TG. We manually checked whether key articles were shown to both reviewers. Subsequently, in order to ensure that we included all relevant articles, the title and abstract of each article that was included by one or both of the reviewers were subjected to a second screening using Rayyan, a web-based tool to streamline and blind the screening process [30]. The rescreening was done by the junior researcher (ST) and two senior researchers (MP, ES). These articles were read and reviewed in full and included when they met the inclusion criteria. Disagreement was resolved through discussion among the reviewers (ST, MP, ES).

### Quality appraisal

The Joanna Briggs Institute (JBI) checklist for critical appraisal of qualitative studies was used to assess the quality of included articles [31, 32]. This widely used checklist consists of 10 questions relating to the quality of qualitative research, which can be answered with ‘Yes,’ ‘No,’ ‘Unclear,’ or ‘Not applicable.’ Two reviewers assessed each article, and disagreement was resolved through discussion with a third reviewer (ST, MP, ES). The quality appraisal was used during the data synthesis by ordering the articles from highest to lowest score, because the sequence can influence the results [33].

### Data extraction and synthesis

Our meta-ethnographic approach [33] aimed to synthesize new themes from the key concepts of included studies and develop new insights into the mechanisms of the lead-up and management of crisis in dementia care. This was achieved by several rounds of determining relations between studies, conducting reciprocal and refutational translations of concepts and synthesizing these translations to develop higher-order interpretations. Below, we describe this process in detail.

One reviewer (ST) extracted data from the studies which included publication details, study population characteristics, methodology and type of crisis. The key concepts from the results sections of the studies were extracted in a ‘meta-ethnographic framework table’, which facilitated finding relations between the results of the different studies. The meta-ethnographic framework distinguishes three levels of data: first order data (original quotations), second order themes (author interpretations) and third order constructs (new categories). First order data preserve the original voices of participants and second order interpretations reflect how the original authors conceptualized those experiences. Third order constructs, developed through synthesis, offer a higher-level, integrative understanding across studies. The complete overview of extracted data in the framework guided the whole analysis process.

First, the framework was discussed with two other reviewers (MP, ES) in one initial three-hour session. During this session, themes from the included studies were assigned the status of axial codes, which enabled the identification of underlying relationships within crisis processes in dementia care. In this session, a previous model of the crisis resolution process which resulted from a qualitative study, guided initial code group categorization as the results of the majority of studies appeared to align with their model [24]. We formulated initial themes, and a draft model depicting the interrelations between themes. After this initial session, the themes and the draft model were compared across studies to validate the model, adjusting the themes and its interrelations iteratively with all authors. We established that the initial formulated themes were strongly interrelated and often centered around the same phenomenon. Hence, in the last phase of analysis, we applied Strauss and Corbin’s coding paradigm [34] to deductively organize the codes enabling us to identify the core phenomenon and map the relationships between themes.

## Results

### Included studies

**Figure 1:**
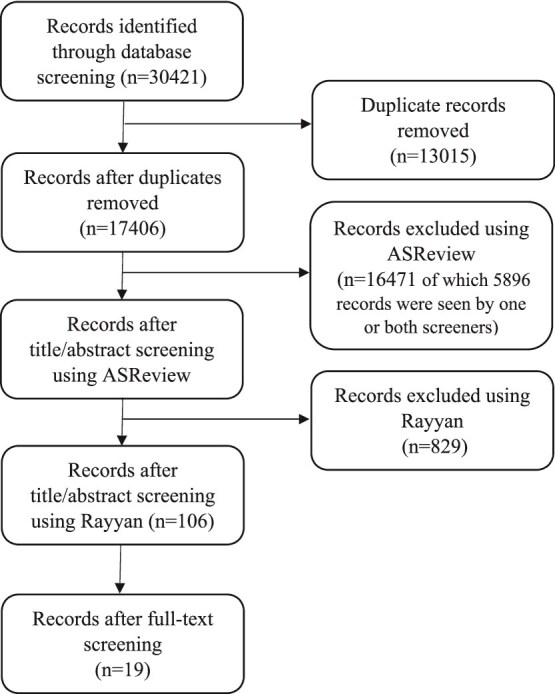
PRISMA Flowchart.

The initial search yielded 30 421 citations of which 17 406 remained after deduplication. Using ASReview, 935 abstracts were included by one or both screeners. These abstracts were reassessed using Rayyan with the additional exclusion criterion ‘studies investigating risk factors for crisis’. After manual rescreening 106 abstracts remained. Full-text screening of these 106 articles resulted in inclusion of 19 qualitative studies. Quantitative articles and articles not related to crisis were excluded (Figure 1).

Table 2 shows characteristics of the included articles, almost half were from the United Kingdom (n = 9) [35–42]. The remaining articles were from the United States (n = 5) [13, 43–46], the Netherlands [47, 48], Germany (n = 1) [49], Australia (n = 1) [50] and Ireland (n = 1) [51]. Few studies included the perspective of people with dementia [24, 35, 36, 39, 47] (n = 5), while most studies included perspectives from healthcare professionals (n = 13) [24, 35, 37, 38, 41–45, 47–49, 51], and informal carers (n = 15) [13, 24, 35–37, 39, 42, 44–51]. One study included members of the public [40]. Some studies explicitly mentioned the term crisis (n = 7) [24, 35–37, 40–42], other articles described ED visits (n = 4) [39, 43–45], hospital admissions (n = 4) [13, 46, 49, 50], long-term care admissions (n = 3) [38, 48, 51] or safety incidents [47]. Of the 19 studies included, four overlapped with the review of Hopkinson et al. [13, 35, 44, 51]. Table 3 shows the JBI quality assessment scores for every article.

**Table 2:** Study characteristics

First author, year	Country	Title	Study design, data collection and analysis	Sample and setting	Aim	Description of crisis
**Beck, 2021**	USA	Features of primary care practice influence emergency care-seeking behaviours by caregivers of persons with dementia: A multiple-perspective qualitative study	Exploratory qualitative study In-person and telephone interviews Thematic analysis	5 primary care providers, 5 geriatrics providers, 4 ED physicians, 5 aging service providers, 3 paramedics, 5 informal caregivers	To explore how primary care features influence emergency care-seeking decisions by people with dementia and their informal caregivers	Emergency care-seeking
**Bosco, 2020**	UK	Narrative inquiry on case studies of crisis in dementia	Case study Interviews over one month Thematic analysis	5 dyads of persons with dementia and informal caregivers	To explore how informal caregivers and people with dementia coped with mental health crises across time	Mental health crisis
**Bosco, 2020**	UK	Involving the Person with Dementia in Crisis Planning: Focus Groups with Crisis Intervention Teams	Exploratory qualitative study Focus groups Framework analysis	22 crisis team staff from 3 NHS Trusts	To explore crisis team perspectives on involving people with dementia in decision-making at all points in the care pathway	Crisis intervention teams
**Cole, 2021**	UK	Professionals' views on the "optimal time" for people living with dementia to move to a care home	Phenomenological qualitative study Interviews Thematic analysis	20 social workers and 20 care home managers	To explore social workers' and care home managers' views on the best time for care home admission for people with dementia	Long-term care admission
**De Jong, 2023**	NL	What Facilitates or Hampers Living at Home With Advanced Dementia Until the End of Life? A Qualitative Study Using Retrospective Interviews Among Family Caregivers, General Practitioners and Case Managers	Qualitative study Interviews Thematic analysis	11 informal caregivers, 2 general practitioners, 9 case managers	To provide insight into factors that support or hamper living at home with advanced dementia until the end of life	Long-term care admission
**Donnelly, 2017**	Ireland	"We do not have the infrastructure to support them at home": How health system inadequacies impact on long-term care admissions of people with dementia	Qualitative study Interviews Thematic analysis and ‘one sheet of paper’ method	22 healthcare professionals, 16 informal caregivers	To understand the role of healthcare system factors in long-term care admissions of people with dementia	Long term care admission
**Groen-Van De Ven, 2016**	NL	Decision Trajectories in Dementia Care Networks: Decisions and Related Key Events	Prospective qualitative study. 3 interviews at 6-month intervals Multilayered analysis using content analysis, affinity diagramming, timeline mapping and constant comparison	23 care networks consisting of 113 people with beginning to advanced stages of dementia and their informal and professional caregivers	To examine the decisions and related key events in the trajectories of care networks including people with dementia, their informal and professional caregivers	Safety incidents
Hopkinson, 2020	UK	Crisis management for people with dementia at home: Mixed-methods case study research to identify critical factors for successful home treatment	Mixed-methods case study Interviews, focus groups and observation. Inductive thematic analysis	5 persons with dementia, 13 informal caregivers and 14 healthcare professionals were interviewed. 15 persons with dementia were observed 9 healthcare professionals participated in a focus group	To identify key factors for successful home-based crisis resolution	Crisis
**Jacobsohn, 2019**	USA	Factors Influencing Emergency Care by Persons With Dementia: Stakeholder Perceptions and Unmet Needs	Qualitative exploratory study Interviews Thematic analysis	4 informal caregivers, 4 emergency medicine physicians, 5 primary care physicians, 5 geriatric healthcare providers, 6 aging service providers and 3 community paramedics	To explore stakeholders’ perspectives on the decisions and drivers influencing emergency department use, and suggestions for effectively addressing unmet needs of persons with dementia	Emergency department use
**Jamieson, 2016**	Australia	Carers: The navigators of the maze of care for people with dementia-A qualitative study	Qualitative study Interviews and focus group Thematic content analysis	30 informal caregivers	To investigate the experiences of people with dementia and their informal caregivers during transition from hospital to home	Hospital admission
**Oliveira, 2019**	UK	Health-Promoting Self-Care in Family Caregivers of People With Dementia: The Views of Multiple Stakeholders	Multimethod, qualitative study Stakeholder consultations and focus groups	46 health and social care professionals, policymakers, informal caregivers and researchers 27 informal caregivers	To explore stakeholder and caregiver views on promoting self-care in dementia caregiving	Crisis
**Pohontsch, 2017**	Germany	(In-)formal caregivers' and general practitioners' views on hospitalizations of people with dementia—an exploratory qualitative interview study	Exploratory qualitative study Interviews Structuring content analysis	12 informal caregivers, 12 general practitioners and 5 healthcare professionals	To investigate circumstances and preventability of hospitalizations of persons with dementia	Hospital admission
**Redley, 2022**	UK	Practitioners' Views on Enabling People With Dementia to Remain in Their Homes During and After Crisis	Qualitative study Interviews Interpretative phenomenological analysis	12 nurses, 1 psychiatrist, 1 occupational therapist and 1 healthcare assistant	To investigate how crisis teams support people with dementia to remain at home	Crisis
**Sadak, 2017**	USA	Potentially preventable hospitalizations in dementia: family caregiver experiences	Qualitative study Interviews Interpretative phenomenological analysis	20 informal caregivers	To describe caregiver experiences during health crises to identify intervention opportunities	Hospital admission after health crisis
**Sharpp, 2016**	USA	Experiences of frequent visits to the emergency department by residents with dementia in assisted living	Mixed-methods study Interviews and focus groups Thematic analysis	9 family members and 3 assisted living employees were interviewed 11 assisted living employees participated in 2 focus groups	To describe the health care incidents leading to ED transfers from assisted living	Emergency department use
**Toot, 2013**	UK	Causes of crises and appropriate interventions: the views of people with dementia, carers and healthcare professionals	Exploratory qualitative study Focus groups Inductive thematic analysis and ‘long table’ approach	18 people with dementia, 15 informal caregivers 19 healthcare professionals	Identified crisis triggers and potential interventions for community-dwelling people with dementia	Crisis
**Williamson, 2023**	UK	Exploring access to community care and emergency department use among people with dementia: A qualitative interview study with people with dementia, and current and bereaved caregivers	Exploratory qualitative study Interviews Experiential reflexive thematic analysis	10 people with dementia, 11 current informal caregivers and 16 bereaved informal caregivers	To explore experiences of accessing community and emergency care for people with dementia	Emergency department use
**Yates, 2020**	UK	Conceptualizing Dementia Crisis and Preferences for Resolution: A Public Perspective	Qualitative study Questionnaire Thematic analysis	57 members of the public	To investigate public views on dementia-related crises and to identify a useful approach for crisis services	Crisis
**Zhou, 2023**	USA	Validation and expansion of a behavioural framework for dementia care partner resilience (CP-R)	Qualitative study Interviews Abductive thematic analysis	20 informal caregivers	To validate and expand the care partner resilience framework by identifying caregivers’ challenges during dementia-related health crises and the behaviours they engaged in response	Hospital admission

**Table 3:** JBI checklist scores

Author, year	JBI checklist item										
	1	2	3	4	5	6	7	8	9	10	Total
**Beck, 2021**	No	Yes	Yes	Yes	Yes	Yes	No	Yes	Yes	Yes	8
**Bosco, 2020**	No	Yes	Yes	Yes	Yes	No	No	Yes	Yes	Yes	7
**Bosco, 2020**	No	Yes	Yes	Yes	Yes	No	No	Yes	Yes	Yes	7
**Cole, 2021**	Yes	Yes	Yes	No	Yes	No	No	Yes	Yes	Yes	7
**De Jong, 2023**	No	Yes	Yes	Yes	Yes	Yes	Yes	Yes	Yes	Yes	9
**Donnelly, 2017**	No	Yes	Yes	Yes	Yes	No	No	Yes	Yes	Yes	7
**Groen-Van De Ven, 2016**	No	Yes	Yes	Yes	Yes	No	No	Yes	Yes	Yes	7
**Hopkinson, 2020**	No	Yes	Yes	No	No	Yes	Yes	Yes	Yes	Yes	7
**Jacobsohn, 2019**	No	Yes	Yes	Yes	Yes	No	No	Yes	Yes	Yes	7
**Jamieson, 2016**	No	Yes	Yes	Yes	Yes	Yes	No	Yes	Yes	Yes	8
**Oliveira, 2019**	Yes	Yes	Yes	Yes	Yes	Yes	Yes	Yes	Yes	Yes	10
**Pohontsch, 2017**	No	Yes	Yes	Yes	Yes	Yes	No	Yes	Yes	Yes	8
**Redley, 2022**	Yes	No	Yes	Yes	Yes	Yes	Yes	Yes	Yes	Yes	9
**Sadak, 2017**	Yes	Yes	Yes	Unclear	Unclear	Yes	No	Yes	Yes	Yes	7
**Sharpp, 2016**	No	Yes	Yes	Yes	Yes	No	No	No	Yes	Yes	6
**Toot, 2013**	No	Yes	Yes	Yes	Yes	No	No	Yes	No	Yes	6
**Williamson, 2023**	Yes	Yes	Yes	Yes	Yes	Yes	Yes	Yes	Yes	Yes	10
**Yates, 2020**	No	Unclear	Yes	Yes	Yes	No	No	Unclear	Yes	Yes	5
**Zhou, 2023**	Yes	Yes	Yes	Yes	Yes	Yes	No	Yes	Yes	Yes	9

### Findings

**Figure 2:**
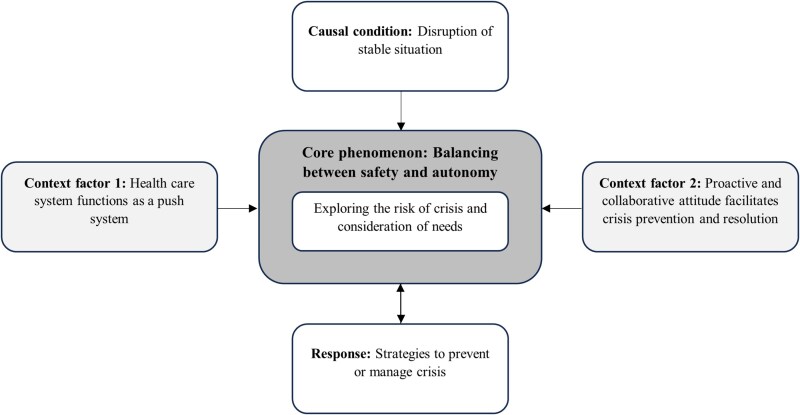
Graphical overview of underlying mechanisms of the lead up and management of crisis situations.

The meta-ethnographic approach yielded five themes describing underlying mechanisms of the lead-up and management of crisis.: a core phenomenon, a causal condition, a response and two context factors. Themes are illustrated by quotes from the original articles. We identified the core phenomenon of *balancing between safety and autonomy,* including exploring the risk of crisis and consideration of needs, which is needed because of disruptions to the current (stable) situation (causal condition). Balancing is described as an active search for an appropriate action strategy to prevent, reduce or resolve the crisis (response) and aiming for acceptable safety while respecting autonomy. Two context factors contribute to the balance between safety and autonomy and thus prevention, reduction or resolution of the crisis: healthcare professionals’ proactive and collaborative attitude (context factor 1) and the health care system that functions as a push system (context factor 2). Figure 2 illustrates the interrelations between themes based on the Strauss and Corbin paradigm. No intervening conditions and consequences were identified in our study.

### Balancing between safety and autonomy

The included articles often describe a crisis by its consequences and the level of care required. Some crises can be managed at home with minor adjustments or sufficient home-based care, while others require hospital or nursing home admission. The core phenomenon that emerged from most included studies was the delicate balancing between safety and autonomy. When dementia progresses and other dementia-related or health issues emerge, persons with dementia were described to face increasing disruptions and risks. Therefore, living at home becomes more hazardous, requiring both persons with dementia and their informal caregivers to weigh the benefits of living at home against the associated risks [38, 47]. Studies indicate that persons with dementia often prefer living at home, which may require the acceptance of safety risks before a crisis [38, 48] and adjusting the level of support when disruptions or crises occur [41, 47, 48].

*‘So, the whole process with the family, they really trusted us. And whatever we gave them in terms of intervention, sometimes not medication, sometimes liaising with the social worker for them, calling them, was a very lengthy process. At the end of the day, they [the family] were aware that things will decline, but they were happy that we sort of took them there slowly.’ (support worker)* [41]

Other than risks of staying at home, studies illustrate that the potentially negative effects of crises resulting in ED visits and hospital admissions are contemplated by healthcare professionals, informal caregivers and persons with dementia themselves [38, 39, 51].

*‘The A&E [Accident and Emergency] isn’t any place for someone with dementia. Hospital isn't any place for someone with dementia. We need to be in our own environment. We need to have hospital at home, if you like, simply because you go in the hospital, you don't come out at the same level as you were when you went in. I have seen it with so, so many of my friends. Just the noise, the lack of routine, lack of knowledge, lack of understanding, makes it just an alien environment. And that's why I simply won't go in anymore.’ (person with dementia)* [39]

Informal caregivers and healthcare professionals strive to respect the person’s autonomy for as long as possible. They describe pushing their own boundaries to enable a person with dementia to stay at home [38, 48].

*‘She [person with dementia] was so vehement about wanting to stay at home that we had to do all in our power to keep her at home, even if it meant things like … she did fall down the stairs, because she refused to put her proper slippers on. We knew that was a risk. But we knew that she actually wanted to stay at home, right the way through. And so, I was very reluctant to [move her]’ (social worker)* [38]

Studies describe that planning for the future is helpful in respecting the autonomy while trying to balance between safety and autonomy [43, 44, 47].

### Exploring the risk of crisis and consideration of needs

In included studies, balancing between safety and autonomy is fueled by exploring the risk of crisis and a consideration of the needs of persons with dementia, their informal caregivers and healthcare professionals. Studies describe key aspects, the most important being the wishes and needs of persons with dementia and informal caregiver, their different experience of disruption, and the resilience of the informal caregiver.

Considering the wishes and needs of the person with dementia and their informal caregiver was described to be essential in deciding which action should be taken to prevent or resolve a crisis [24, 37, 38, 41, 48]. Studies indicate that the wishes of persons with dementia are sometimes overlooked and timely discussions about the future are emphasized to support including these wishes in decision-making.

*‘And we need to be brave enough to say to people, “look you know you could drop dead with a heart attack tomorrow, but if your dementia progresses you'll live long enough and it has an impact on your life, what would you like [to happen]?”’(care home manager)* [38]

The challenge of balancing safety and autonomy around a disruption is mentioned in several studies to be complicated by the different experiences people with dementia, informal carers and healthcare professionals have regarding a crisis and they may even disagree about whether a situation is a crisis [36, 37, 40, 41, 44, 46].

*‘It was OK for me, full stop. No crisis. [about a situation a carer did view as crisis].’ (person with dementia)* [36]*.*

When the person with dementia or their informal caregiver do not perceive a situation as a crisis, this may prevent them from seeking or accepting help [36, 37]. The opposite is also described when informal caregivers feel they need to convince healthcare professionals to act [46]. Different healthcare professionals involved may also interpret the needs of the person with dementia and their caregiver differently, which can lead to ineffective care [37].

*‘Very often we get a referral from the GP and when we go out we see that they did not need us at all. So yeah the definition of crisis is very different for everyone’ (crisis intervention team staff)* [37]

Studies describe that perceptions of crisis vary depending on the nature of the disruption, the severity of dementia symptoms and the personal characteristics of the person with dementia [36, 41].

*‘it depends on so many other factors like family, friends, what support [they are receiving], their level of dementia, and most importantly what they want.’ (social worker)* [38]

Healthcare professionals frequently describe informal caregiver resilience as an essential part of risk assessment. Informal caregivers express the burden of constantly monitoring their partner, lacking personal time and experiencing sleep problems, but also coping strategies like accepting the situation and help-seeking [42, 46]. Studies indicate that health and social professionals assess the resilience of informal carers by evaluating their physical health, coping mechanisms and the support of the wider network [13, 35, 46, 48–50].

*‘I have seen a lot of situations where I’ve said ‘the home situation is no longer workable’. But I reckon it depends mainly on the caregiver and whether they can cope. […] And I went all-out to support him, as I thought he would be able to cope.’ (case manager)* [48]

### Causal condition: disruption of the stable situation

In the context of crisis, one or more disruptions that increase risks to a person with dementia or their environment cause the need for balancing between safety and autonomy. In the included papers, the nature of these disruptions was mentioned to be acute or to develop more gradually. Acute and unexpected disruptions that immediately lead to a crisis were identified. For example, when the person with dementia (or informal caregiver) acquires an infection or falls [13, 35, 38, 40, 48, 49]:

*‘She would lay in her bed and she would just see things and talk about things that weren’t there, I got frightened and took her to the hospital, turned out she had bad UTI. (informal caregiver)* [13]

Other crises were mentioned to result from a buildup of slower, smaller disruptions [38, 47]. These changes can include progression of dementia symptoms such as forgetfulness [35] and communication problems [49]; behavioural problems such as agitation and wandering [13, 35, 48, 49]; and slowly progressing health problems such as mobility problems and incontinence [46, 49]. Additionally, caring for the person with dementia becomes more challenging for their informal carers and may gradually lead to caregiver overburden [13, 35, 38, 42, 46, 48].

*‘Their [person with dementia's] risks are 'bubbling'. […..] If they are starting to 'bubble' and family are anxious, it needs to kind of tip before, it then becomes a high risk to then say “okay, being at home is no longer [an option].’ (social worker)* [38]

All these changes lead to the balance shifting to more risks and the need for new strategies to respond to the disruption.

### Response: strategies to prevent and manage a crisis

Studies describe that after exploring the risks of the disruption, a decision is made for a strategy to manage the situation and hopefully prevent or resolve the crisis. Studies indicate that the nature of the crisis, along with the personal and contextual factors of the person with dementia and their informal caregiver, influences whether a disruption can be resolved before a crisis occurs [40, 48].

In some cases, crises may be prevented or resolved at home. Studies suggest that having care and support in place, such as sufficient home care and a trusted, long-term case manager or care team, can help prevent disruptions from developing into crises [35, 39, 48]. Additionally, supporting the informal caregiver by offering day care, respite care and informal caregiver education is considered crucial to prevent or resolve a crisis at home.

*‘Quite often [there is] reassurance [in] having somebody like myself turn up, talk through things, put things into perspective, and point out perhaps a few changes that the carer could make in how they are providing care for their husband, wife, or whomever. I always found that actually, that went a [long] way in resolving what you would call a crisis.’ (health care professional)* [41]

Many studies describe that resolving disruptions at home, although preferred, is not always possible. In some cases, a crisis cannot be resolved at home necessitating an ED visit or hospital admission [13].

*‘Definitely, yes, definitely. There are situations where a person with dementia falls or becomes ill with something that the nursing home or the private home environment cannot evaluate or are not equipped to deal with. I am completely convinced that I’d be the person to 100% support sending these people to the hospital quickly even if a doctor just quickly takes a look to make sure everything’s ok […].’ (informal caregiver)* [13]

Healthcare professionals describe these different action strategies as being aimed at prevention or resolution of the crisis by restoring the situation to a stable state. This is a situation with an optimal balance between risk and safety for the person with dementia and their informal caregiver(s).

### Context factor 1: proactive and collaborative attitude facilitates crisis prevention and resolution at home

Included studies suggest that the process of thorough balancing and adapting to a new situation towards and during a crisis is facilitated if healthcare professionals have a proactive and collaborative attitude. Studies indicate that crises can more easily be resolved in the primary care setting if the healthcare professionals involved are familiar with the situation of persons with dementia and their caregivers [43].

*‘If it hasn’t been discussed prior to an acute episode, a person is going to choose more care, more intervention, more transfers—more. Because there’s that fear aspect that it hasn’t been discussed. The not knowing just prompts people to “do”. The only way I can see a person reducing the “let’s just do it blindly and go forward” is to talk about ahead of time.’ (primary care professional)* [43]*.*

The process of balancing and adapting is described to be facilitated by an interprofessional care team that maintains effective communication between professionals, e.g. through multidisciplinary team assessments [35, 43]. Individuals in the network know each other and can refer people to suitable support at the right time [44].

Findings indicate that a proactive attitude among healthcare professionals is closely linked to their familiarity with the person with dementia and their informal caregivers. This familiarity often involves investing in a trusting and long-term relationship [13, 24, 36, 37, 41, 43].

*‘someone [health care professional] you can rely on that you trust you can get hold of. I know not everybody is available 24 hours a day.’ (informal caregiver)* [35]

Studies describe that proactive help can come from either a dedicated professional or a care team that is well-acquainted with the situation. In these cases, effective communication between healthcare professionals is described as crucial for understanding the situation thoroughly by sharing important needs and issues [24, 37, 41, 43].

*‘There could be a team of three or four people involved … that includes the primary care physician. He or she directs a couple of people that are gonna be the team for this person, and you are gonna call your team prior to calling the ambulance or taking them to the ED if you have issues. That way, a few people can learn about that person very intricately.’ (paramedic)* [43]

Studies also describe that a proactive attitude includes knowing the right resources to help informal caregivers navigate the system to prevent crisis [35, 43, 44, 49]. Being aware of available resources facilitates choosing the right action strategy that takes specific risks and needs of a person into account.

### Context factor 2: health care system functions as a ‘push system’

In contrast to the lower level of organization, in which a ‘proactive and collaborative attitude facilitates crisis prevention and resolution’, many system level factors are described as factors that hinder preventing or managing crisis. When a person with dementia and their informal caregiver do not receive the right care and support at the right time due to insufficient resources, crisis situations were described to occur more frequently and often lead to ED visits and hospital admissions [39, 51]:

*‘…when something is going poorly, having nothing in place to get help in the home is why the ER has become such as common last refuge for these patients. I think they feel like there’s nowhere else they can go.’ (geriatric healthcare provider)* [44]

Health care services are frequently described as fragmented, under-resourced or overstretched [37, 39, 45, 51] making them difficult to access:

*‘I just think all the services are under stress in terms of resources and they basically all try to push things to other services because they do not have enough time to manage the cases all by themselves.’ (crisis intervention team staff)* [37]

Several health care system elements were identified as barriers to receiving timely and appropriate care, thereby increasing the risk of disruptions that could lead to ED visits or hospital admissions during acute situations. These elements include health care services and social services lacking funding or staff to give the needed care or support [38, 41, 51], no available emergency beds [51], no integrated system between facilities [44], no primary contact point [42], no primary care available outside office hours [43]. Sometimes, home care or nursing home care is reported to be easier to arrange after a hospital admission or ED visit [39, 51].

When the healthcare system is fragmented and under-resourced, individuals often adapt their behaviour to navigate it, experiencing it as a ‘push system.’ Healthcare services appear to push people towards other services, while individuals themselves must become ‘pushy’ to secure appropriate care and support [39, 50]. Many studies have highlighted the lack of integration and communication between services as a key factor making the healthcare system difficult to navigate. [39, 42–44, 46].

*‘That the whole support for people with dementia it's much like, I suppose you could argue it's not a pull system, it's a push system. And it relies very much on the support that those people are receiving because, you know, to get the best treatment and help for them. Yeah, it's not a pull system. You have to get very pushy. It's quite hard I think to navigate.’ (informal caregiver)* [39]

## Discussion

### Study summary

This meta-ethnography of the lead-up and management of crisis in persons with dementia identified several underlying mechanisms, with ‘balancing between safety and autonomy’ standing out as the core phenomenon. Studies included describe disruptions that destabilize the situation of the community-dwelling person with dementia, and lead to increased risks, as drivers for this balancing between safety and risk. In crisis situations, consideration of needs of the person with dementia and informal caregiver enables healthcare professionals to choose suitable action strategies to resolve the situation. This process of balancing between safety and risk contributes to prevention or appropriate management of crises. Significant contextual factors in this process were: a proactive and collaborative attitude of healthcare professionals that can facilitate, and the existing healthcare system that frequently functions as a push system and hinders the process.

### Comparison with previous reviews

Building on Hopkinson et al.'s review [52], we sought to advance the understanding of the mechanisms behind the crisis process that was described by Hopkinson et al. Since their review, insights into crisis have expanded, as reflected by the 10 additional articles published after 2019 that our search strategy identified. Four articles overlapped with this review [13, 35, 44, 51], others differed due to a different operationalization of crisis in our review, allowing for an expanded analysis. We chose to exclusively include qualitative studies aimed at understanding mechanisms behind the crisis process in contrast to the more exploratory set-up including quantitative and qualitative studies in the review by Hopkinson et al. Our findings align with Hopkinson’s work with regards to key elements of crisis like the disruption of a stable situation, the need for risks assessment and actions that lead to risk management, although in Hopkinson’s review these elements are not explicitly defined as themes. Our study further refines these concepts emphasizing balancing as an active response to disruptions, incorporating risk assessment and the individual needs of people with dementia. The core phenomenon of balancing between safety and autonomy shows that crisis is a non-linear process unlike Hopkinson’s description (before—during—after), which matches the crisis definition by Vroomen in which crisis is seen as a process triggered by stressors that create imbalance, requiring immediate decisions to achieve resolution; otherwise, the cycle continues [8]. Our crisis model integrates this cyclical understanding of crisis of Vroomen et al. [8] with the understanding that crisis consists of several phases described by Hopkinson et al. [52] By emphasizing the underlying mechanisms of crisis, our study adds actionable insights.

### Impact of contextual factors on lead-up and management of crisis

While Hopkinson et al. described the phases of crisis management in home-based dementia care, our study expands on their work by explicitly outlining two interrelating context factors: a facilitating proactive and collaborative attitude and hindering health care system factors in shaping crisis prevention and resolution.

We found that a proactive and collaborative approach from healthcare professionals facilitates effective crisis management and prevention for people with dementia. This includes actively engaging with informal caregivers and anticipating potential stressors before they escalate, which is consistent with literature about successful collaboration in dementia care [53] and which is part of crisis interventions [54, 55]. Myhre et al. previously and similarly described how a proactive attitude, as opposed to a reactive approach, facilitates crisis prevention in frail older people [56].

Our study found that healthcare system factors, such as high staff turnover, fragmented services and inadequate resources, contribute to the occurrence of crises in dementia care. These findings are consistent with broader evidence from primary care, where health care system fragmentation and inadequate primary care organization have been shown to lead to hospitalizations in older people [57, 58] and persons with dementia in particular [59]. These findings underscore how systemic shortcomings not only compromise care delivery, which has been described in a recent review [60], but also increase vulnerability to crisis in dementia care.

### Strengths and limitations

The most important strength of this study was the use of meta-ethnography methodology combining both inductive and deductive analyses that facilitated a more in-depth understanding of the mechanisms that underlie the lead-up and management of crisis in dementia care. The heterogeneity of the research team, including several clinicians, enhanced the analytical depth and ensured that multiple perspectives were integrated throughout the research process. Another strength was the use of ASReview, which allowed for a sensitive search strategy fitting the exploratory aim. As ASReview is a relatively new tool that depends on algorithms, the selection process lacks transparency. Therefore, we incorporated additional safeguards, including duplicate screening and a second title/abstract screening in Rayyan, to ensure robustness and reduce potential bias in study selection. As the included studies were conducted in diverse healthcare systems, differences in the organization of dementia care may limit the generalizability of findings. Nevertheless, systems factors were largely similar across countries.

### Implications

The emergence of ‘active balancing between safety and autonomy’ as a core phenomenon in dementia care, particularly during disruptions, provides a perspective of action to crisis prevention and management. Dementia-specific skills training can support healthcare professionals in assessing the risk of crisis and identifying the needs of people with dementia and their informal caregivers, particularly during periods of instability or disruption. In cases of gradual deterioration, a practical tool to support early recognition of potential crises, e.g. an approach similar to the 'surprise question' used in palliative care, may contribute to anticipate clinical decline and guide proactive care planning. The two contextual factors, a proactive and collaborative attitude that facilitates crisis prevention and resolution and the health care system that may function as a ‘push system,’ can serve as key targets to develop interventions to prevent or manage crisis situations. A collaborative and proactive attitude may be achieved by interventions aimed at improving integrated care, which are currently being developed [61, 62]. Whether these interventions will help prevent crises should be studied further.

Health policy should prioritize ensuring sufficient, well-trained staff and take concrete actions to reduce structural fragmentation in the healthcare system. This can be achieved through better care coordination and appropriate funding mechanisms that support integrated service delivery.

### Overall conclusion

This meta-ethnography provides a better understanding of the mechanisms underlying the process towards and during crisis in dementia care and suggests possible interventions to improve care and prevent crises. By identifying the process of balancing between safety and autonomy and facilitating and hindering contextual factors, it highlights concrete opportunities to enhance professional practice through proactive and collaborative care approaches and to guide policy reforms aimed at reducing fragmentation and improving continuity of care.

## Supplementary Material

aa-25-1834-File002_afaf383
