# Supplementary material for: From bubbling to boiling over: a meta-ethnography of the process towards and during crisis from the perspectives of persons living with dementia, informal carers and healthcare professionals
Source: Age Ageing. 2026 Jan 24;55(1):afaf383. doi: 10.1093/ageing/afaf383 (PMC13156077; doi:10.1093/ageing/afaf383)
Supplement: aa-25-1834-File002_afaf383 [file aa-25-1834-file002_afaf383.docx]

**From bubbling to boiling over: A meta-ethnography of the process towards and during crisis from the perspectives of persons living with dementia, informal carers, and healthcare professionals**

# Appendix 1: Search strategy

## **Pubmed**

(dementia[Mesh] OR dement*[tiab] OR Alzheimer*[tiab] OR "Frontotemporal lobar degeneration*"[tiab] OR "Lewy body disease"[tiab] OR FTLD[tiab])

AND

(emergency[tiab] OR crisis*[tiab] OR crises[tiab] OR "Crisis intervention"[Mesh] OR Hospitalization[Mesh:NoExp] OR hospitalization*[tiab] OR (Admission[tiab] AND (hospital[tiab] OR patient[tiab] OR acute[tiab] OR nursing home [tiab])) OR "Patient Admission"[Mesh] OR "Emergency Medical Services"[Mesh])

AND

(2000:2023[pdat])

## **PsycInfo**

(exp Dementia/ OR dement*.tw OR Alzheimer*.tw OR "Lewy Body disease".tw OR "frontotemporal lobar degeneration".tw OR FTLD.tw)

AND

(Emergency Services/ OR emergency.tw OR * Crisis Intervention/ OR "crisis*" OR crises OR * Hospitalization/ OR hospitalization*.tw OR * Hospital Admission/ OR "hospital admission*".tw OR "patient admission*".tw OR "acute admission*".tw OR "nursing home admission*".tw)

Filtered for articles between 2000 and 2023

## **Embase**

(exp dementia/ or dement*.ti,ab,kf. or Alzheimer*.ti,ab,kf or "frontotemporal lobar degeneration".ti,ab,kf or ‘Lewy Body disease*’.ti,ab,kf.)

AND

(emergency/ or "emergency*".ti,ab,kf or crisis*.ti,ab,kf or crises*.ti,ab,kf or crisis intervention/ or hospitalization/ or "hospitalization*".ti,ab,kf or hospital admission/ or "hospital admission*".ti,ab,kf or "patient admission*".ti,ab,kf or "acute admission*".ti,ab,kf or "nursing home admission*".ti,ab,kf)

Filtered for articles between 2000 and 2023

## **Web of science**

(dement*. or Alzheimer or frontotemporal lobar degeneration" or "Lewy Body disease*" or “FTLD”)

AND

(emergency* or "crisis" or "crises" or "hospitalization*" or "hospital admission*" or "patient admission*" or "acute admission*" or "nursing home admission*")

Filtered for articles between 2000 and 2023

## **Cinahl**

((MH "Dementia+") OR (TI "dement*" OR AB "dement*") OR (TI “frontotemporal lobar degeneration” OR AB “frontotemporal lobar degeneration”) OR (TI “FTLD” OR AB “FTLD”)OR (TI “lewy body disease*” OR AB “lewy body disease*”) OR (MH "Dementia Patients"))

AND

((MH "Hospitalization of Older Persons") OR (MH "Patient Admission") OR (MH "Hospitalization") OR (TI “hospitalization*” OR AB “hospitalization*”) OR (TI “hospital admission*” OR AB “hospital admission*”) OR (TI "patient admission*" OR AB “patient admission*”) OR (TI "acute admission*" OR AB “acute admission*”) OR (TI "nursing home admission*" OR AB “nursing home admission*”) OR (MH "Emergency Service") OR (TI “emergency” OR AB “emergency”) OR (MH "Crisis Intervention") OR (TI “crisis” OR AB “crisis”) OR (TI “crises” OR AB “crises”))

Filtered for articles between 2000 and 2023

## **Cochrane**

(MeSH descriptor: [Dementia] explode all trees OR (dement*):ti,ab,kw OR (Alzheimer*):ti,ab,kw OR ("lewy body disease"):ti,ab,kw OR ("Frontotemporal lobar degeneration"):ti,ab,kw OR ("FTLD"):ti,ab,kw)

AND

(MeSH descriptor: [Emergencies] this term only OR MeSH descriptor: [Crisis Intervention] explode all trees OR MeSH descriptor: [Hospitalization] this term only OR MeSH descriptor: [Patient Admission] explode all trees OR (emergency):ti,ab,kw OR (crisis*):ti,ab,kw OR (crises):ti,ab,kw OR (hospitalization*):ti,ab,kw OR (hospital NEXT admission*):ti,ab,kw OR (patient NEXT admission*):ti,ab,kw OR (acute NEXT admission*):ti,ab,kw OR ("nursing home" NEXT admission*):ti,ab,kw

AND

Cochrane Library publication date Between Jan 2000 and Dec 2023
